# Genome-Wide Identification, Characterization, and Expression Analysis of Long-Chain Acyl-CoA Synthetases in *Carya illinoinensis* under Different Treatments

**DOI:** 10.3390/ijms241411558

**Published:** 2023-07-17

**Authors:** Wenjuan Ma, Kaikai Zhu, Juan Zhao, Mengyun Chen, Lu Wei, Zhenbing Qiao, Pengpeng Tan, Fangren Peng

**Affiliations:** 1 Co-Innovation Center for Sustainable Forestry in Southern China, Nanjing Forestry University, Nanjing 210037, China; mabuer1998@163.com (W.M.); kkzhu@njfu.edu.cn (K.Z.); cxyl@njfu.edu.cn (J.Z.); hehecmy@163.com (M.C.); wl18236975076@163.com (L.W.); qzb1227@163.com (Z.Q.); tanpengpeng2002@163.com (P.T.); 2 College of Forestry, Nanjing Forestry University, Nanjing 210037, China

**Keywords:** pecan, long-chain acyl-CoA synthases, expression analysis, subcellular localization, qRT-PCR

## Abstract

As crucial enzymes in the lipid metabolic network, long-chain acyl-CoA synthases (LACSs) are members of the acyl-activated enzyme superfamily and play a crucial role in epidermal wax synthesis, plant lipid anabolic metabolism, and stress tolerance. In this study, 11 pecan *LACS* genes were identified and categorized into five groups and located on nine chromosomes. The significant degree of conservation in the AtLACS and CiLACS protein sequences was demonstrated by multiple sequence alignment and conserved motif analysis. Cis-acting element analysis identified numerous stress-responsive and hormone-inducible elements in the promoter regions of *CiLACS* genes. The expression levels of *CiLACS9* and *CiLACS9-1* were considerably up-regulated under salt and drought stress, according to the qRT-RCR study. Treatment with ABA also led to increased expression levels of *CiLACS1*, *CiLACS1-1*, *CiLACS2*, and *CiLACS9-1*. Notably, *CiLACS4*, *CiLACS4-1*, *CiLACS9*, and *CiLACS9-1* exhibited peak expression levels at 135 days after anthesis and are likely to have been crucial in the accumulation of seed kernel oil. Moreover, the *CiLACS9* gene was shown to be located in the cytoplasm. These findings offer a theoretical framework for clarifying the roles of *LACS* genes in the processes of pecan kernel oil synthesis and response to abiotic stressors.

## 1. Introduction

The acetyl coenzyme A synthetase (ACS) superfamily, which may be further subdivided into short-chain, medium-chain, long-chain, and extra-long-chain lipid coenzyme A synthases based on the length of their carbon chains [1], includes long-chain acyl-CoA synthetases (LACSs). They are crucial in the production of plant lipids. The synthesis of lipids in plant epidermal waxes and seeds requires the participation of the substrate lipid coenzyme A [2], LACS catalyzes the formation of lipid coenzyme A from free fatty acids in a two-step reaction, in which the free fatty acids react with ATP in the presence of Mg^2+^ to form the adenosylated intermediate acyl-AMP, which then binds to the thiolipid bond of CoA to release lipid coenzyme A and AMP [3]. The AMP-binding domain in LACS, which is highly conserved and specific to the AMP-binding superfamily, and a conserved ACS signaling motif in the AMP-binding superfamily both play important roles in this activation process [4,5]. LACS can contribute significantly to plant stress tolerance and fatty acid anabolic metabolism thanks to these particular structural configurations.

Several members of the LACS family in plants were found in various subcellular compartments, including the endoplasmic reticulum, peroxisomes, and the chloroplast outer membrane. They were involved in crucial processes like fatty acid metabolism, lipid biosynthesis, waxy keratin biosynthesis in the plant epidermis, and fatty acid catabolism [6,7,8]. Nine *LACS* genes (*AtLACS1*~*AtLACS9*) have been found in *Arabidopsis thaliana*, and these genes were involved in various aspects of life [7,9]. *AtLACS3* is involved in keratin formation and was highly expressed in dry epidermal cells, while *AtLACS1* and *AtLACS2* have some overlapping functions in epidermal lipid metabolism and are involved in wax synthesis [10,11]. *AtLACS6* and *AtLACS7* were localized in the peroxisome and are involved in fatty acid oxidation [12]. *AtLACS9* was designated in plastids and can act in conjunction with the biosynthesis of triacylglycerols in Arabidopsis seeds [13]. In addition, LACS genes were identified in kale oilseed rape (*Brassica napus*), where *BnLACS4* was highly expressed in flowers and developing seeds of kale-type oilseed rape and increased the lipid content of a yeast strain [14], while inhibition of *BnaLACS8A03* expression led to a reduction in thioglycoside content in kale oilseed rape. Sequence similarities between *HaLACS2* and *HaLACS1* from sunflower (*Helianthus annuus*) and *AtLACS8* and *AtLACS9* from *Arabidopsis* have been found. While *HaLACS2* was tightly bound to the endoplasmic reticulum and may be involved in sunflower lipid synthesis, HaLACS1 protein was highly expressed in the outer membrane of tobacco (*Nicotiana benthamiana*) chloroplasts [6]. Members of the *LACS* gene family contribute significantly to the response to abiotic stresses and the synthesis of plant seed oils. *MdLACS1* in apple (*Malus pumila*) increased the healing tissue’s tolerance to polyethylene glycol, salt, and abscisic acid, demonstrating that *MdLACS1* is a key regulator in apple response to abiotic stresses [15]. After treatment with NaCl, PEG, and ABA, the majority of *HaLACS* genes in sunflower significantly increased in expression level [16]. In soybean (*Glycine max*), *GmLACS2-3* overexpression lines significantly increased keratin and lignin content, and they also showed greater drought tolerance [17]. In addition, *GmLACS9/15/17* were significantly up-regulated under alkali, low-temperature, and drought stresses [18]. *OsLACS9* is a designated protein in the chloroplast envelope of rice (*Oryza sativa*), and its dysfunction causes massive starch deposition in the chloroplast and plant dwarfing [19].

The pecan (*Carya illinoinensis* [Wangenh.] K. Koch) is a species of economic tree belonging to the genus *Carya* in the family Juglandaceae that is indigenous to America [20]. With an oil yield of up to 70% and a content of 90% unsaturated fatty acids, the seed kernels are high in protein and fatty acids. In actuality, the proportion of polyunsaturated fatty acids (PUFAs) to saturated fatty acids (SFAs) in pecan seeds is very similar to that of olive oil [21] Unsaturated fatty acids can lower blood levels of cholesterol and triglycerides, regulate heart function, and provide human health benefits [22]. The pecan tree is also a good timber and ornamental tree with its tall shape and beautiful crown; therefore, it has a high economic value. However, numerous biotic and abiotic stresses frequently have an impact on the pecan crop’s production value. According to previous studies [15,18,23], the *LACS* gene was found to play an important role in lipid synthesis and catabolism in plants, as well as in resistance to abiotic stresses, and is important for the selection and breeding of thin-shelled pecan varieties to increase yield and efficiency. However, the *LACS* gene in pecan has not yet been identified or extensively studied. In this study, 11 *CiLACS* genes were found across the entire genome. A better understanding of the molecular mechanisms underlying lipid accumulation and stress tolerance in pecan kernels will be made possible by the additional analysis of the evolutionary relationships, tissue-specific expression, response to abiotic stresses, and expression patterns of the CiLACS family during various stages of kernel development.

## 2. Results

### 2.1. Genome-Wide Identification and Characterization of LACS Genes in Pecan

Eleven LACS genes were selected from the whole pecan genome; they were renamed (CiLACS1~CiLACS9-1) based on their similarity to *Arabidopsis*, and their amino acid sequences were examined. According to the findings, the *CiLACS* genes encode amino acid residues with a theoretical range of 613 to 730, the theoretical relative molecular masses of the encoded proteins range from 67.60 to 880.16 kDa, the theoretical isoelectric points are 5.96~8.36, and the theoretical instability coefficients are 27.79~40.36 (Table S1). All others had instability coefficients below 40, except for *CiLACS2*, which had an instability coefficient of 40.36. According to the results of the predicted subcellular localization, six CiLACS proteins were located in the chloroplast, one was located in the nucleus, and four were located in the cytoplasm.

**Figure 1:**
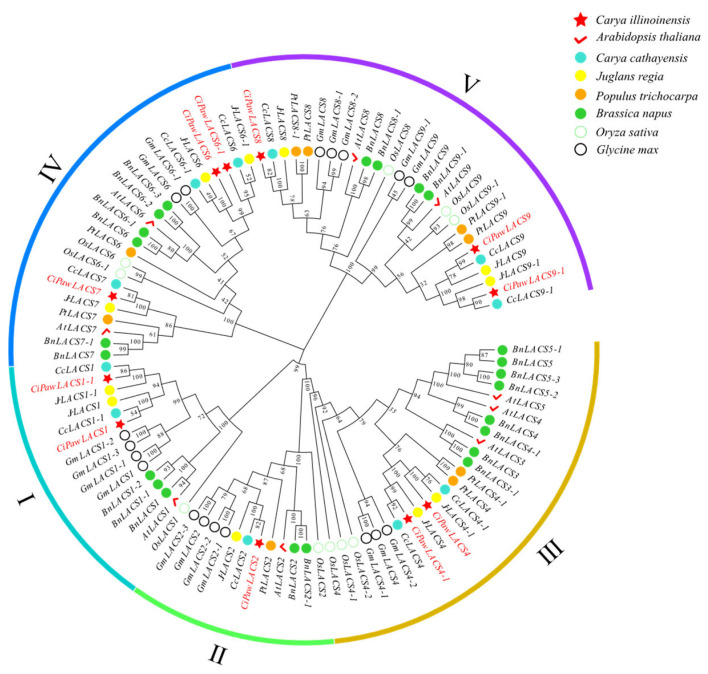
Phylogenetic analysis and classifications of the LACS proteins in pecan and 7 other plant species. The species are distinguished by different colored shapes. LACS from six species were divided into five groups (groups I, II, III, IV, and V). The groups are highlighted in different colors.

LACS protein sequences from pecan and seven additional species (*Arabidopsis*, rape, Chinese hickory, soybean, poplar, and rice) were used to create phylogenetic trees, and LACS members of these species were renamed in accordance with their affinities to *Arabidopsis* (Table S2). The results revealed that the CiLACS proteins were more evenly distributed among the five subclades of LACS proteins, which formed a total of five subclades (Figure 1). Of them, three belonged to subclade 5, two to subclade 1, one to subclade 1, two to subclade 2, two to subclade 3, and three to subclade 4. These CiLACSs, however, demonstrated a stronger affinity for pecan.

### 2.2. Multiple Sequence Alignment, Gene Structure, and Conserved Motif Analysis of the CiLACS Genes

**Figure 2:**
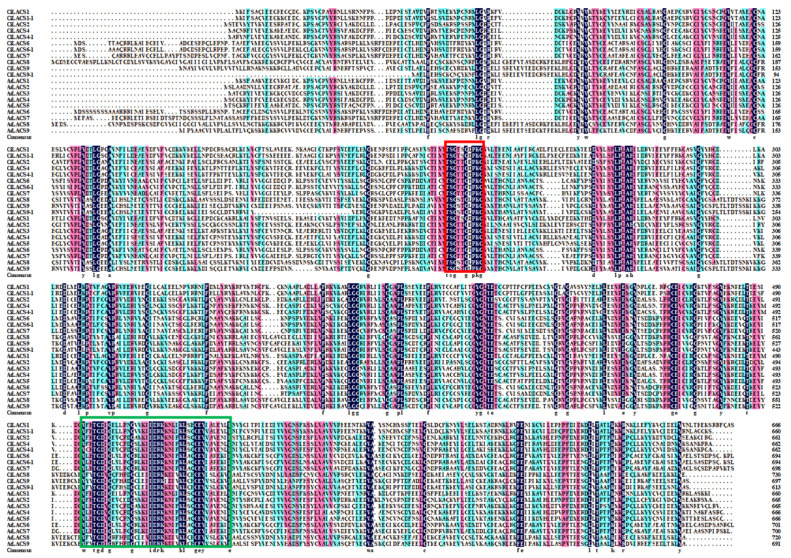
Multiple sequence alignment of CiLACS and AtLACS proteins. Red boxes represent AMP-binding structural domains and green boxes represent ACS signaling structural domains.

Eleven pecans and nine *Arabidopsis* had high homology and high conservation in their LACS amino acid sequences (Figure 2). The AMP-binding domain (red box) and the ACS signaling domain (green box), two commonly conserved structural domains of the LACS family, are shared by nearly all members of the CiLACS family.

**Figure 3:**
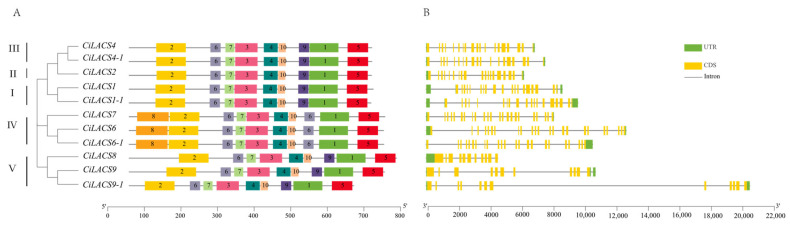
The gene structure (**A**) and conserved motifs (**B**) of *CiLACS* family genes encoding proteins. The horizontal axis of (**A**) indicates the length of the protein sequence, and the horizontal axis of (**B**) indicates the length of the promoter sequence.

Gene structure and motif analyses of the CiLACS genes were carried out to examine the evolutionary conservation of these genes. The results revealed that CiLACS has roughly the same number of exons and introns within the same subfamily, with some variation between subfamilies (Figure 3A). In subclades 1, 2, and 3, there are 19 exons and 18 to 20 introns, while in subclade 4, there are 23 exons and 22 introns, and in subclade 5, there are 11 exons and 10 to 11 introns. The CiLACS proteins are highly conserved throughout evolutionary history, as shown by a motif analysis of the CiLACS protein (Figure 3B), which revealed that each CiLACS contains an AMP-binding domain (motif 3) and the ACS signaling domain (motif 1).

### 2.3. Chromosomal Localization and Colinearity Analyses of CiLACS Genes

**Figure 4:**
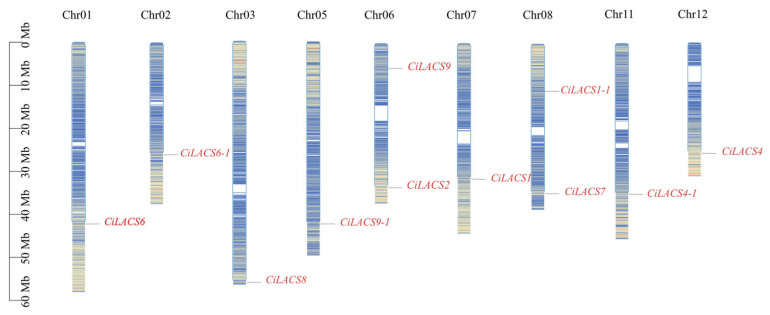
Chromosomal localization of the *LACS* genes in pecan. *CiLACS* genes are marked in red, and each chromosome’s dense lines depict how all of the genes on that chromosome are distributed.

Using the MCScanX program, which is based on the genomic database, the intraspecific homology of 11 *CiLACS* genes and the interspecific homology of 9 *Arabidopsis LACS* genes were examined to better understand the potential functions of *CiLACS* genes. The results are shown in Figure 4. All of the identified *CiLACS* genes were found to be located on the nine pecan chromosomes. Each chromosome had one to two *CiLACS* genes, with chromosomes 6 and 8 having the most. The pecan LACS family also contained a total of six colinear pairs and fifteen colinear pairs with the model plant *Arabidopsis*, with each member of the family sharing at least one homologous pair of genes with *Arabidopsis* (Table S3).

Ka and Ks were further calculated to analyze the traits of CiLACS family members during evolution. *Ka/Ks* can be used to explore the selective pressure on genes during evolution; a value of *Ka/Ks* > 1 denotes positive selection, a value of *Ka/Ks* < 1 denotes negative selection, and a value of *Ka/Ks* = 1 denotes neutral selection. The replication genes of the pecan *LACS* family had *Ka/Ks* values between 0.1 and 0.2, which suggests that the *CiLACS* genes underwent significant negative selection during evolution (Table S4).

### 2.4. Tissue-Specific Expression Analysis of the CiLACS Genes

**Figure 5:**
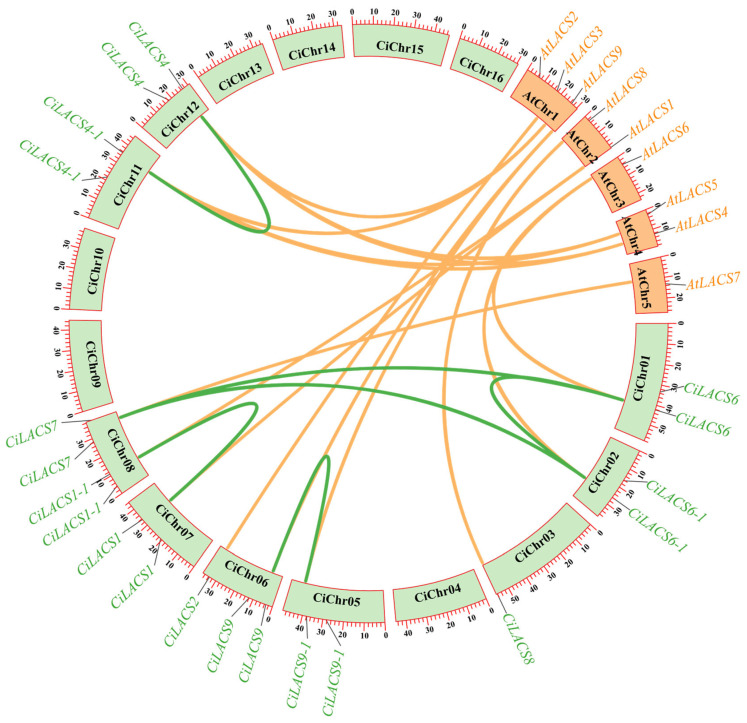
Schematic representations of the interchromosomal relationships and chromosomal locations of *CiLACS* and *AtLACS* genes. The green lines in the circle indicate the duplicated *LACS* gene pairs in the pecan, and the orange lines indicate duplicated *LACS* gene pairs in *Arabidopsis*. The *CiLACS*s are marked in green, and the *AtLACS*s are marked in orange. The chromosome number is indicated in the middle of the chromosomes filled with green and orange.

**Figure 6:**
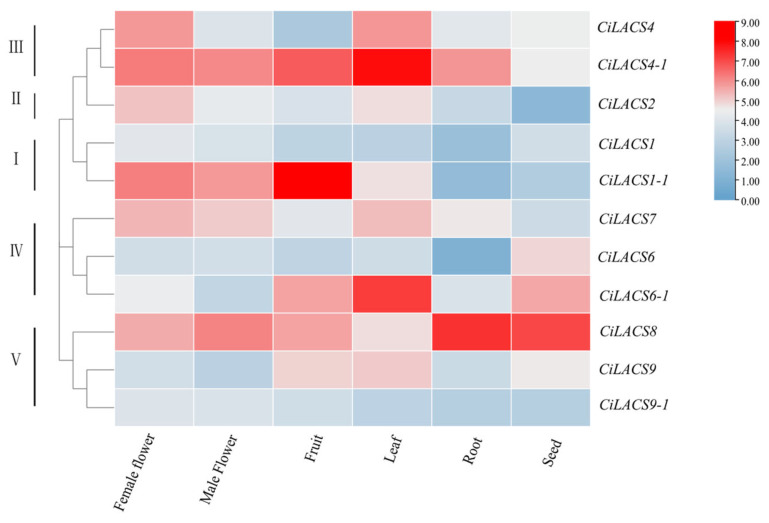
Expression abundance of *CiLACS* genes in different tissues.

Understanding the biological functions of genes is aided by tissue-specific analysis of those genes. Analysis of transcriptome data based on pecan in six different tissues (Figure 5) to determine the tissue expression levels of *LACS* genes revealed that *CiLACS4-1* was expressed at high levels in all tissues except seeds; *CiLACS8* was expressed at high levels in all tissues, especially in roots and seeds; *CiLACS1-1* was expressed at high levels in flowers and fruits; *CiLACS6-1* and *CiLACS9* were expressed at high levels in fruits, leaves, and seeds; and *CiLACS1* and *CiLACS9-1* were highly expressed in flowers and fruits (Figure 6).

### 2.5. Cis-Element Analysis in the Promoter Regions of the CiLACS Genes in Pecan

**Figure 7:**
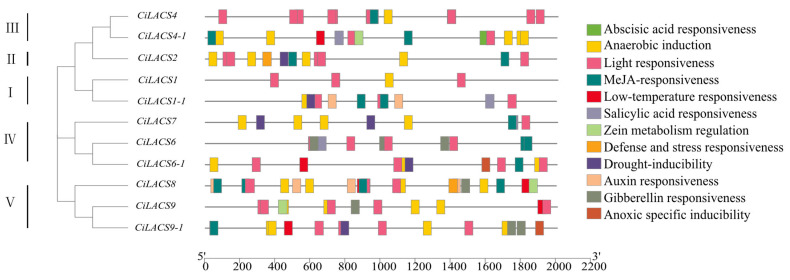
Cis-element analysis in the *CiLACS* gene promoter regions. Three types of cis-acting elements were identified, including hormone-responsive elements, elements related to resistance to harsh environments and stresses, and elements related to plant growth and development.

Potential cis-acting elements on the *CiLACS* gene promoter regions were analyzed to forecast the putative functions of the *CiLACS* genes in response to various abiotic stresses. The findings (Figure 7) demonstrated that all 11 *LACS* gene promoter regions contained numerous hormone- and environment-like response elements, with the highest concentration among the hormone-like response elements (ABA, methyl jasmonate, salicylic acid, etc.) being that of ABA response elements. Additionally, there are numerous light response elements in the *CiLACS* gene promoter region.

### 2.6. Expression Analysis of CiLACS Genes under Different Stress Conditions and during Different Periods of Fruit Development

**Figure 8:**
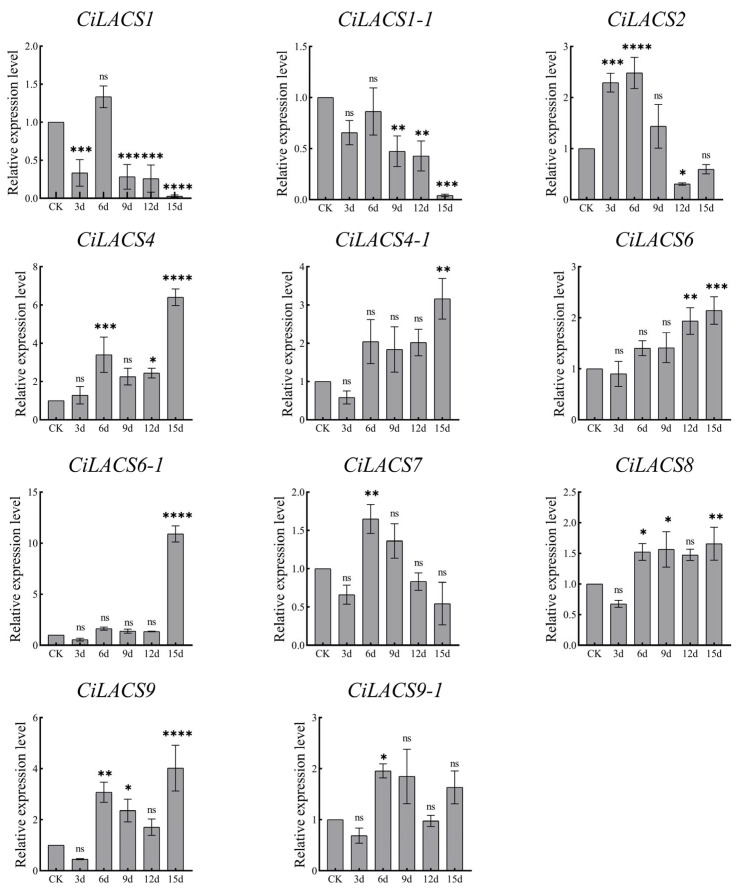
Relative expression of *CiLACS* genes under drought treatment. The reference gene is the actin gene (*CiPaw.03G124400*). * *p* < 0.05, ** *p* < 0.01, *** *p* < 0.001, **** *p* < 0.0001.

**Figure 9:**
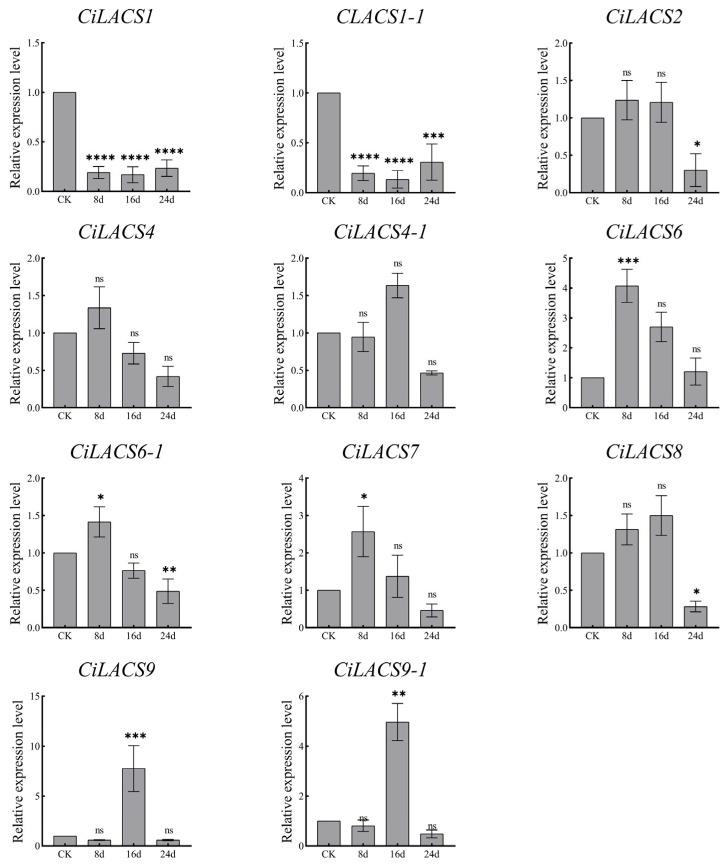
Relative expression of *CiLACS* genes under NaCl treatment. The reference gene is the actin gene (*CiPaw.03G124400*). * *p* < 0.05, ** *p* < 0.01, *** *p* < 0.001, **** *p* < 0.0001.

**Figure 10:**
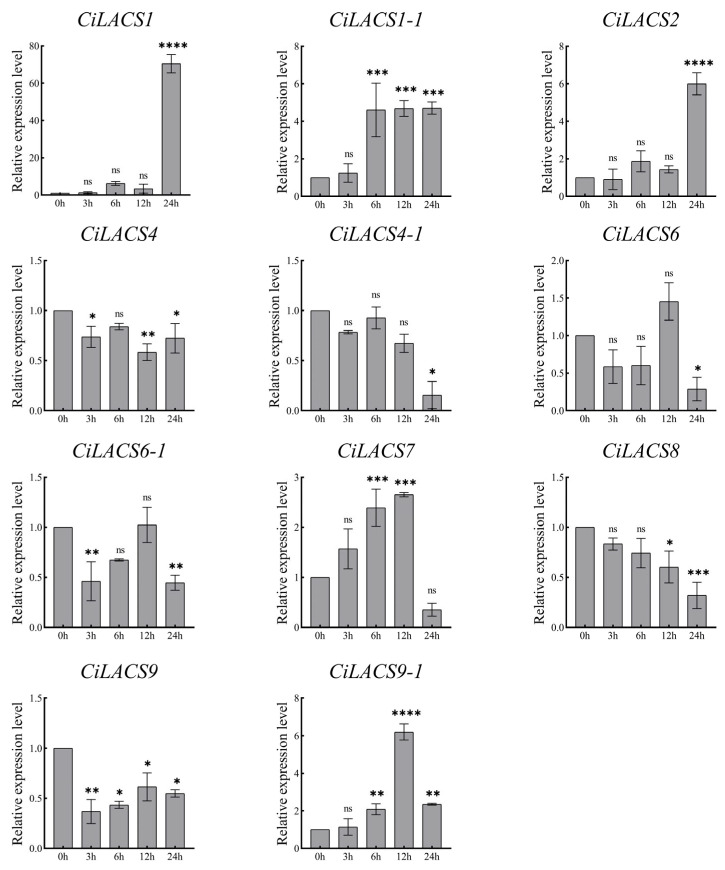
Relative expression of *CiLACS* genes under ABA treatment. The reference gene is the actin gene (*CiPaw.03G124400*). * *p* < 0.05, ** *p* < 0.01, *** *p* < 0.001, **** *p* < 0.0001.

We examined the levels of *CiLACS* expression in pecan under drought, NaCl stress, and ABA induction to examine the response of 11 *CiLACS* genes to abiotic stress and hormone induction (Figure 8, Figure 9 and Figure 10), and the findings revealed that the majority of *CiLACS* genes responded to the various treatments. *CiLACS4*, *CiLACS4-1*, *CiLACS6-1*, and *CiLACS9* expression levels were significantly up-regulated during the drought treatment (*p* < 0.05), peaking 15 days after treatment, whereas *CiLACS1*, *CiLACS1-1*, *CiLACS2*, and *CiLACS7* expression levels were down-regulated as the drought treatment progressed. *CiLACS9* and *CiLACS9-1* expression levels peaked on day 16 after treatment, while *CiLACS1*, *CiLACS1-1*, and *CiLACS4-1* expression levels were down-regulated in salt stress. The expression levels of the other genes peaked on day 8 after NaCl treatment before declining. Several *CiLACS* genes, including *CiLACS1*, *CiLACS1-1*, *CiLACS2*, *CiLACS7*, and *CiLACS9-1*, had increased expression levels in response to ABA treatment. In particular, the expression level of *CiLACS1* was 70-fold higher at 24 h of treatment than it was at 0 h.

**Figure 11:**
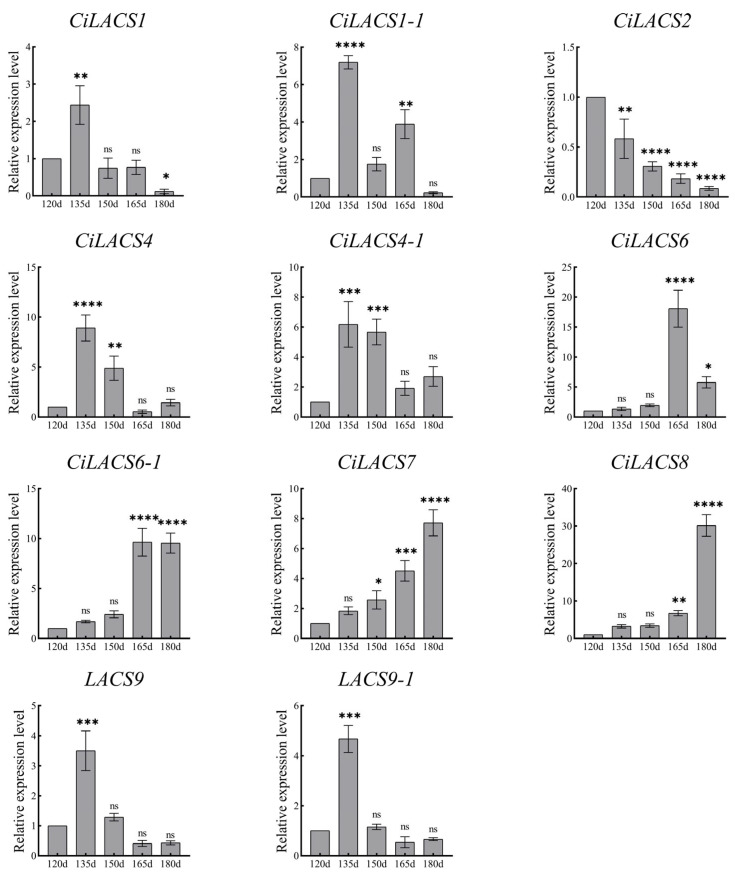
Relative expression of *CiLACS* genes at different developmental stages of seed kernels. The reference gene is the actin gene (*CiPaw.03G124400*). * *p* < 0.05, ** *p* < 0.01, *** *p* < 0.001, **** *p* < 0.0001.

We also examined the expression of *CiLACS* in pecan fruits at five different times to further understand the expression of *LACS* genes during oil synthesis in pecan fruits. The outcomes confirmed what was anticipated (Figure 11). Nearly all of the CiLACS genes were up-regulated; *CiLACS1*, *CiLACS1-1*, *CiLACS4*, *CiLACS4-1*, *CiLACS9*, and *CiLACS9-1*, as well as *CiLACS6*, *CiLACS6-1*, and *CiLACS7*, reached their peak expression levels 135 days after flowering before being down-regulated. The expression levels of *CiLACS6*, *CiLACS6-1*, *CiLACS7*, and *CiLACS8* were still up-regulated 120 days after flowering and peaked 165 and 180 days after flowering.

### 2.7. Subcellular Localization of CiLACS9s in Tobacco Leaves

**Figure 12:**
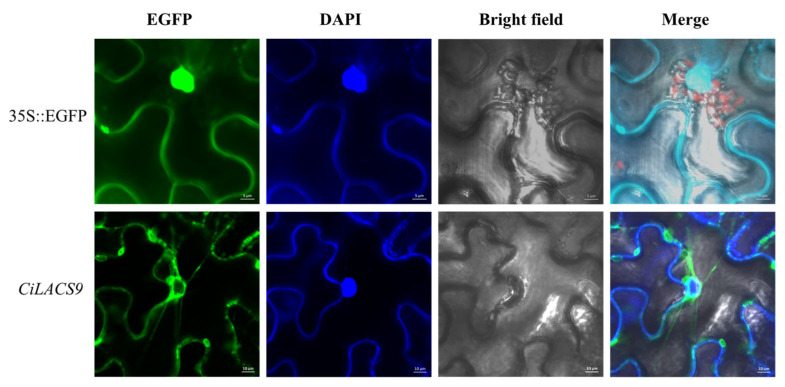
Subcellular localization of CiLACS9 in lower epidermal cells of tobacco leaves. The vector 35S::GFP was used as the control. Bar = 10 μm, bar = 5 μm.

LACSs play a role in many biological processes involving fatty acids, and understanding their subcellular localization can shed light on their functional functions [13,24]. In this study, EGFP fluorescence of *CiLACS9* fusions was mainly detected in the cytoplasm and wrapped around the periphery of the nucleus, as shown by subcellular co-localization experiments under confocal laser microscopy (Figure 12).

## 3. Discussion

With the advancement of high-throughput sequencing technology in recent years, an increasing number of researchers have been studying some important gene families linked to particular plant traits and biological functions at the whole-genome level. Preliminary research has been conducted on the structure and function of *LACS* genes that have been isolated from plants including *Arabidopsis*, rice, wheat, and kale-type oilseed rape. Although *LACS* genes have been demonstrated to be crucial for plant lipid synthesis and stress resistance [25], there is currently a lack of comprehensive reports on the identification and functional studies of this gene family in pecan. In this study, we identified 11 members of the *CiLACS* gene family in pecan and divided them into five subfamilies (Figure 1). Multiple sequence alignment of *CiLACS* and *AtLACS* showed that *CiLACS* protein sequences have AMP-binding (PF00501) and ACS signaling domains typical of the *LACS* family (Figure 2). Notably, AMP binding plays an important role in lipid anabolic metabolism, which was consistent with previous results in *Arabidopsis* [26]. Interestingly, the conserved motifs of *CiLACS* family members within the same subfamily showed high similarity (Figure 3A,B). Subfamilies I, II, and III also showed high similarity in the type, number, and position of motifs, with subfamily IV being slightly different from the other subfamilies, having motif 9 in addition to motif 8 but not motif 6. Additionally, the number of introns–exons in the *CiLACS* family is relatively consistent across subclades, suggesting that *LACS* genes are evolutionarily conserved and *LACS* genes within subclades may have similar functions.

Gene duplication plays a significant role in the amplification of gene families during evolution, and adaptation to the environment through the family members acquired in response to selection pressure [20]. Chromosomal localization showed that (Figure 4) the 11 *CiLACS* genes were distributed on nine chromosomes, with *CiLACS2* and *CiLACS9* on the same chromosome and *CiLACS1-1* and *CiLACS7* on the same chromosome. The different chromosomal distributions suggest that pecan *LACS* genes have undergone genetic variation during evolution. Colinearity and *Ka/Ks* (Table S4) analyses showed that there were 15 colinear gene pairs between pecan *LACS* and *Arabidopsis LACS*, and six colinear gene pairs in pecan *LACS*, and these genes were all derived from fragment duplication, indicating that fragment duplication played a central role in the evolution of *CiLACS* (Figure 5). Surprisingly, the *Ka/Ks* values of the *CiLACS* colinear gene pairs were all less than 1, suggesting that *CiLACS* family genes may have been subject to strong negative selection during evolution (Table S4). During gene evolution, repetition time may lead to the accumulation of degenerative mutations, whereas negative selection may favor the evolution of the family genes by eliminating the deleterious variants that emerge, which also suggests that the *CiLACS* family genes are functionally conserved.

It has been discovered that the promoter regions of *CiLACS* genes also contain a number of regulatory elements associated with hormone response and stress, including ABA, MeJA, drought, and low-temperature response elements, the presence of which may be crucial in the ability of CiLACS to enhance resistance in pecan [27]. Previous studies have demonstrated that LACS proteins are involved in the activation of long-chain fatty acids and produce lipid acyl-coenzyme A, which is crucial for plant growth and development. *AtLACS1, AtLACS2*, and *AtLACS4* are crucial for the production of keratin and waxes that make up the epidermis in *Arabidopsis*. Among them, double mutants of *AtLACS1* and *AtLACS2* have decreased epidermal wax content and increased epidermal permeability, while mutants of *Arabidopsis AtLACS1* and *AtLACS4* have 35% and 73% of the wax content of the wild type, respectively [9]. The physiological and metabolic regulation of epidermal wax in *Arabidopsis* helps plants cope with drought, salt, and ABA treatment [28,29,30]. These studies suggested that the mechanism of the response of *LACS* genes to environmental stress may be due to their regulatory role in wax synthesis. Here, we investigated the expression patterns of *CiLACS* family genes in different pecan tissues and under different treatments (Figure 6). Comparison of *CiLACS* expression levels in different tissues revealed that most *CiLACS* genes showed high expression levels in flowers and leaves, and it was hypothesized that these genes might be involved in the synthesis of epidermal waxes in flowers and leaves. The expression levels of *CiLACS4-1*, *CiLACS6-1*, *CiLACS8*, and *CiLACS9* were higher in most tissues, especially in fruits and mature seeds, and it is speculated that these genes may be involved in plant seed oil synthesis. Furthermore, the *CiLACS* genes showed different expression patterns under different treatments. Fifteen days after the no-watering treatment, the expression levels of the genes *CiLACS4*, *CiLACS4-1*, *CiLACS6*, *CiLACS6-1*, and *CiLACS9* were consistently up-regulated; in particular, the relative expression of *CiLACS6-1* on day 15 was 10 times higher than that of CK. Under salt stress, the expression levels of *CiLACS6*, *CiLACS7*, *CiLACS9*, and *CiLACS9-1* peaked 8 and 16 days after treatment. Under ABA treatment, the relative expression of *CiLACS1*, *CiLACS1-1*, and *CiLACS2* peaked at 24 h of treatment, and *CiLACS7* and *CiLACS9-1* peaked at 12 h post-treatment; in particular, the relative expression of *CiLACS1* was more than 60-fold higher at 24 h than it was at 0 h. These results indicate that these genes play a crucial role in pecan in response to ABA, drought, and salt treatments.

Fatty acids are produced in the plastid of the plant’s body, where they are activated to lipoyl CoA before entering the endoplasmic reticulum to take part in TAG synthesis. Previous studies revealed that *AtLACS1* and *AtLACS2* were located in the endoplasmic reticulum, and the *AtLACS1*, *AtLACS2* double mutant caused a reduction in the production of leaf epidermal wax [9]. *BnLACS4* was also localized in the endoplasmic reticulum and may be involved in lipid synthesis in kale-type oilseed rape [14]. *AtLACS9* was designated in the chloroplast envelope. Associated with LACS activity in chloroplasts [13]. *OsLACS9* in rice was localized in the plastids of onion epidermal cells, and its mutation resulted in the accumulation of large amounts of starch granules in chloroplasts [19]. The subcellular localization of *CiLACS9* in this study showed that *CiLACS9* was located in the cytoplasm, wrapped around the nucleus, and formed a network-like structure. Previous studies have shown that *LACS* genes are involved in plant seed oil synthesis. In this study, the expression patterns of *CiLACS* genes during pecan seed kernel development from five different developmental stages were analyzed, and it was found that the expression levels of *CiLACS1*, *CiLACS1-1*, *CiLACS4*, *CiLACS4-1*, *CiLACS9,* and *CiLACS9-1* peaked 135 days after flowering (high oil accumulation stage), while *CiLACS6*, *CiLACS6-1*, *CiLACS7*, and *CiLACS8* peaked 165 days after flowering (stable oil synthesis period). These *CiLACS* genes displayed different expression patterns in different stages of kernel development, indicating that they were crucial for lipid synthesis and metabolism at various stages of kernel development.

## 4. Materials and Methods

### 4.1. Genome-Wide Identification of the LACS Gene in Eight Different Plant Species

The well-studied sequences of nine *Arabidopsis* (*Arabidopsis thaliana*) LACS proteins [1] were downloaded from The *Arabidopsis* Information Resource (TAIR, http://www.Arabidopsis.org/index.jsp (accessed on 6 November 2022)). To identify the *LACS* family members from the entire pecan (*Carya illinoinensis*) genome, we downloaded the Hidden Markov Model (HMM) file of the AMP domain (AMP-binding: PF00501) from Pfam (http://pfam.xfam.org/ (accessed on 6 November 2022)) [31] and used as a query to investigate *LACS* in pecan using HMMER software (version 3.1b2) (www.hmmer.org (accessed on 6 November 2022)). The candidate LACS proteins were further examined by BLASTP analysis [32] in the NCBI database (https://blast.ncbi.nlm.nih.gov/Blast.cgi (accessed on 6 November 2022)), and redundant sequences were removed from the list. Furthermore, the same identification method was used to identify the *LACS* in Chinese hickory (*Carya cathayensis*), rice (*Oryza sativa*), poplar (*Populus trichocarpa*), walnut (*Juglans regia*), rape (*Brassica napus*), and soybean (*Glycine max*).

### 4.2. Physicochemical Characterization of CiLACS Family Protein Sequences

Isoelectric points (PIs), molecular weights (MWs), numbers of amino acids, and instability indexes were predicted using the ExPASY ProtParam [33] server (https://www.expasy.org/resources/protparam (accessed on 8 November 2022)).

### 4.3. Multiple Sequence Alignment and Phylogenetic Analysis

The multiple sequence alignment of the 11 CiLACS proteins and 9 AtLACS proteins was carried out using DNAMAN software (version 7.0) with default parameters (http://dnaman.software.informer.com/ (accessed on 9 November 2022)). The evolutionary relationships among the LACS protein sequences of pecan and seven other species were established by constructing a phylogenetic tree via the neighbor-joining method (NJ method) with the following settings: bootstrap method, 1000 replications; Poisson model and complete deletion, using MEGA software (version 7.0) [34]. The phylogenetic tree consisted of several species, including *Arabidopsis thaliana*, Chinese hickory, rice, poplar, walnut, rape, and soybean. The resulting tree was visualized via the online website Evolview (https://evolgenius.info/ (accessed on 9 November 2022)) [35].

### 4.4. Analysis of Gene Structure and Conserved Motifs

Gene structure information of pecan *LACSs* was retrieved by surveying the General Feature Format (GFF) file from GigaDB (http://gigadb.org/dataset/100571 (accessed on 12 November 2022)) [36]. The information about the gene structure diagram of the 11 *CiLACS* genes was generated using TBtools software (https://github.com/CJ-Chen/TBtools (accessed on 12 November 2022)) [37]. Conserved motifs of *CiLACS* protein sequence were identified using the MEME program (http://meme-suite.org/tools/meme (accessed on 12 November 2022)) [38] with the search number of 10 and the optimum motif length of between 6 and 100 residues.

### 4.5. Chromosome Localization and Gene Duplication Event Analysis among CiLACSs and AtLACSs

The chromosomal distributions of 11 *CiLACS* genes and 9 *AtLACS* genes were visualized using the Circos program in TBtools software. Colinearity analysis of the *CiLACS* and *AtLACS* was performed using the MCScanX program in TBtools software. Similarly, colinearity analysis between *CiLACS* genes was also carried out by this method. To detect the selection pressure of the duplication events, the CDSs of the *CiLACS* and *AtLACS* were aligned using TBtools software, and the synonymous substitution (Ks) and nonsynonymous substitution (Ka) of tandem and segmental duplication events were calculated using MEGA software (version 7.0) [39]. Finally, the selection pressure was evaluated using the *Ka/Ks* ratios.

### 4.6. Tissue-Specific Expression Analysis of CiLACS Gene Family

To further characterize the different expression patterns of the *CiLACS*s in various organs, the transcriptome data were downloaded from accession numbers GSE179336 and PRJNA799663 that were previously generated by our group and include root, leaf, mature pistillate and staminate flower, young fruit, and seed. The expression levels of all *CiLACS* genes were quantified based on their fragments per kilobase per million of reads mapped (FPKM) values using RSEM software [40]. The results, log_2_ expression values of the 11 *CiLACS* genes, were used for heat-map generation using TBtools software (version 7.0).

### 4.7. Cis-Acting Element Analysis

We downloaded the entire pecan genome sequence from the Phytozome database (https://phytozome-next.jgi.doe.gov/ (accessed on 15 November 2022) and extracted the 2000 bp genomic sequence upstream of the transcription start site of these 11 *CiLACS* genes. Then, the cis-acting elements were predicted using PlantCARE software (version 7.0, https://phytozome-next.jgi.doe.gov/ accessed on 15 November 2022) [41]. The hormone response-related elements in the 2000 bp upstream regions of *CiLACS* genes were visualized using TBtools software (version 7.0).

### 4.8. Plant Material and Sample Collection

The material used in this experiment was obtained from the pecan experimental base of Nanjing Forestry University (119°9′6″ E, 31°52′45″ N). For the drought treatment, the annual grafted pecan variety “Pawnee” was moved to the climatic chamber of No. 403, Biotechnology Building, Nanjing Forestry University, in August 2020, and three groups of three plants were put up. The incubation conditions were 14 h of light at 24 °C, 10 h of darkness at 22 °C, 60~70% humidity, watering twice a week, and a 15-day drought treatment (no watering) after one month. Leaves were collected on days 3, 6, 9, 12, and 15 of the drought treatment. For the ABA and NaCI treatments, annual pecan seedlings were transported to the climatic chamber of Nanjing Forestry University, Biotechnology Building, No. 403, in June 2022, in groups of three plants, with three groups. The humidity ranged from 70% to 80%, and the temperature ranged from 25 to 22 degrees. Watering was performed every 2 days, and ABA and NaCI treatments were administered after one week. Leaves were collected on days 8, 16, and 24 after NaCI treatment and at hours 3, 6, 12, and 24 after ABA treatment. Pecan fruit was harvested from six healthy 9-year-old plants of pecan variety “Pawnee” in 2022 on days 120, 135, 150, 165, and 180 after flowering; it was stored on ice, with the skins were peeled off and the seed kernels retained.

Three biological replicates were set up for each sample collected. The collected leaves and seed kernels were immersed in liquid nitrogen for quick freezing, and then they were kept in a −80 °C refrigerator.

### 4.9. Subcellular Localizations of the CiLACS9 Protein

To determine the subcellular localizations of the *CiLACS9s* and the design of primer sequences for cloning *CiLACS9* using snapgene software (version 7.0, Table S5), the full-length sequences of the *CiLACS9s* were inserted into 35S::EGFP vectors, and the resulting constructs were transformed into the *Agrobacterium* strain GV3101 [42]. Then, the leaves of *Nicotiana benthamiana* were used as the host for colocalization experiments involving the *Agrobacterium*-mediated transient expression of the target gene. After injection of the plasmid carrying the target gene into the leaves, the plants were grown normally for 2 days after incubation in the dark at 22 °C for 24 h. The leaves were cut out and placed in DAPI dye for 10 min, and then the lower epidermis of the leaves was observed using a laser scanning confocal microscope (Zeiss LSM710). Excitation wavelengths for EGFP and DAPI were 488 nm and 405 nm.

### 4.10. RNA Collection and Real-Time Quantitative PCR (qRT-PCR)

All collected samples were ground into powder in liquid nitrogen, and total RNA was extracted according to the instructions for the RNA extraction kit (Vazyme, Nanjing, China). cDNA was subsequently synthesized by reverse transcription using a reverse transcription kit (Vazyme, Nanjing, China), stored in a −20 °C refrigerator, and set aside. Primers for 11 *CiLACS* genes were designed using the online website Primer Quest Tool (https://sg.idtdna.com/PrimerQuest/Home/Index (accessed on 20 September 2022) and were synthesized by Beijing Prime Tech Biotechnology Co. (Beijing, China) (Table S6).

PCR amplification was performed using cDNA as a template and β-actin (*CiPaw.03G124400*) as the internal reference gene [34]. The total volume of the qRT-PCR reaction system (Vazyme, Nanjing, China) was 20 μL, including SYBR 10 μL, cDNA (120 ng/μL) 1 μL, upstream and downstream primers 0.4 μL each (10 μmol/μL), and ddH2O 8.2 μL. The reaction procedure was as follows: pre-denaturation at 95 °C for 30 s; denaturation at 95 °C for 10 s, annealing at 60 °C for 30 s, extension at 72 °C for 30 s. A total of 30 cycles were performed. The relative gene expression was calculated using the 2^−ΔΔCT^ method [43].

### 4.11. Statistical Analysis

Statistical analysis was performed using IBM SPSS25 software (SPSS Inc., Chicago, IL, USA). One-way analysis of variance (one-way ANOVA) and multiple comparisons (LSD Duncan) were selected for significance analysis (*p* < 0.05).

## 5. Conclusions

In conclusion, we discovered 11 *LACS* genes in pecan and divided them into five subgroups. The evolutionary relationships, gene structures and conserved structural domains, chromosomal positions, duplication events, and expression patterns of the *CiLACS* family of genes were investigated. The findings imply that genes from the *CiLACS* family may be crucial for pecan oil synthesis and stress resistance. These discoveries significantly advance our knowledge of *LACS* genes in pecans and lay the groundwork for future investigations into the role of *LACS* family genes in pecans and other species.

## Data Availability

Not applicable.

## Supplementary Materials

The following supporting information can be downloaded at: https://www.mdpi.com/article/10.3390/ijms241411558/s1.
